# Exploring physical literacy in school contexts: a systematic review of qualitative evidence

**DOI:** 10.3389/fspor.2025.1713780

**Published:** 2025-12-17

**Authors:** Daniel Vieira, Teresa Silva Dias, Dean Dudley, Paula Batista

**Affiliations:** 1 Centre of Research, Education, Innovation and Intervention in Sport (CIFI2D), Faculty of Sport, University of Porto, Porto, Portugal; 2 Centre for Research and Intervention in Education (CIIE), Faculty of Psychology and Educational Sciences, University of Porto, Porto, Portugal; 3 School of Human Movement and Nutrition Sciences, University of Queensland, St Lucia, QLD, Australia

**Keywords:** physical literacy, physical education, qualitative synthesis, school-based interventions, teaching-learning strategies

## Abstract

**Background:**

Physical Literacy (PL) is widely acknowledged in international policy documents for its holistic contribution to promoting lifelong physical activity. It encompasses four interconnected learning domains (physical, psychological, cognitive, and social), thus extending beyond motor competence. Although theoretical developments in PL have advanced, qualitative insights into its implementation and experience within school-based interventions remain limited. Existing reviews have largely focused on quantitative outcomes, often reinforcing the centrality of the physical learning domain while overlooking the cognitive, psychological, and social dimensions of PL.

**Purpose:**

This study builds on previous reviews by mapping qualitative data on school-based PL interventions. The goal was to deeply understand how PL is developed and experienced in authentic educational contexts.

**Methods:**

Following the PRISMA 2020 guidelines, a three-stage search strategy was conducted across seven databases (EBSCOhost, ProQuest, Cochrane, PubMed, ScienceDirect, Scopus, Web of Science). Eligibility criteria for the review required full-text studies on: PL interventions; conducted in school settings; targeting individuals aged 5–18 years; reporting qualitative results; and written in English, Portuguese and Spanish. Twenty-one studies met the eligibility criteria and were analysed using a hybrid thematic approach, combining deductive and inductive approaches.

**Findings:**

The thematic analysis yielded three themes: i) elements and pedagogical strategies related to the four domains of physical literacy; ii) challenges to program implementation; and iii) recommendations for practice. Results reveal an emphasis on the psychological domain, particularly aspects like motivation, confidence, and engagement, with less attention given to the physical and social domains. Nevertheless, many interventions effectively integrated pedagogical strategies that fostered multiple domains simultaneously, reinforcing PL's holistic nature. Student-centred learning, ipsative assessment, autonomy-supportive teaching, and peer collaboration were consistently linked to positive outcomes across domains.

**Conclusions:**

The review offers insight into how PL is operationalised and experienced in schools. Four main conclusions emerged: i) students engage more deeply with flexible, inclusive, and individualised programmes; ii) student-centred methods and non-judgemental environments are particularly well received; iii) the teacher's role is central to successful implementation; and iv) continuous professional development and collaborative opportunities for teachers are essential for sustaining high-quality PL practices.

## 1 Introduction

The United Nations SDGs 2030 (1) include a global commitment to achieve universal health coverage and to reduce inequalities in access to health services across all countries. In this context, policy initiatives that promote physical activity for everyone are consistent with the promotion of health as a universal right and an essential resource for daily life (2). It is here that Physical Literacy (PL) emerges as a concept for achieving these goals, given that the WHO, in its Global Action Plan on Physical Activity 2028–2030 (see Strategic Objective 3: Action 3.1), highlights the importance of fostering PL to encourage lifelong engagement in physical activity.

The prevalence of discussion around PL in the literature has increased in recent decades, reflecting a broadening understanding of holistic health and lifelong engagement with movement (3), becoming a central theme in curriculum guidelines and policy documents worldwide (4–8). Many of these documents are philosophically grounded in Whitehead's (9–11) work and widely adopted definition of PL: “*the motivation, confidence, physical competence, knowledge, and understanding to value and take responsibility for engagement in physical activities for life”* (12). This definition is informed by complex philosophical underpinnings, particularly those of phenomenology, existentialism and monism (10, 13, 14).

However, whilst the consensus regarding the importance of PL has been growing, its enacted conceptualization remains contested, with multiple definitions and interpretations coexisting in the literature (6, 12, 15), and the search for consensus seems to be an “endless academic debate” [(16); p.1]. PL is interpreted differently across geographical regions, as cultural (social and political) influence appears to be decisive in assigning meaning to the term (17, 18). Moreover, the definition of PL varies depending on the field of application (e.g., health, physical activity, sports, education…) (19–21).

Beyond considerations of geographical context and specific research domains, scholars have identified predominant perspectives on PL that reflect the ongoing debate surrounding its conceptualisation and assessment. These approaches are commonly characterised along a spectrum of idealist to pragmatic perspectives (21, 22): i) the idealist perspectives argues that PL is a holistic and indivisible construct, therefore, its domains (physical, cognitive, affective, and social) cannot be looked at and measured separately. Researchers aligned with this perspective usually explore qualitative methodologies to explore the subjective, lived experiences of individuals engaging in movement (13, 21); ii) the pragmatic perspectives argue for a measurable and operationalised approach, emphasising the need for empirical evidence to change current practices. Pragmatists often use quantitative methods to track progress across distinct domains of PL, making it more adaptable to large-scale assessments and policy implementation (21, 23); iii) the “idealist-pragmatic” perspective has emerged, advocating for a balanced approach that acknowledges the holistic nature of PL while recognising the necessity of practical assessment tools [e.g., the Green Cluster proposed by Young et al. (21)]. This perspective combines theoretical ideals and empirical application, integrating mixed methods to capture both qualitative experiences and quantitative measures of PL (23, 24).

Nevertheless, whilst ongoing conceptual debate continues to attract attention, significant research and support has emerged that recognises PL encompasses the learning undertaken across the physical, cognitive, social and affective learning domains (25, 26). To provide conceptual clarity within educational contexts, this review adopts an operational definition aligned with Keegan et al. (27), viewing PL as lifelong learning expressed through movement experiences: demonstrated when students apply movement skills, make informed decisions, regulate emotions, and interact constructively with others during physical activity. This perspective acknowledges both the holistic nature of PL and the practical ways in which it becomes observable in school settings.

This operational perspective aligns naturally with educational environments. In the field of education, Whitehead (11) argues that PL is not an alternative to Physical Education (PE); PE represents a disciplinary area of the school curriculum, while PL should be seen as the goal of PE. In this sense, PE is often identified as a key setting for fostering PL due to its emphasis on inclusive, movement-based learning experiences (25, 28). This perspective is supported by international policy documents, such as UNESCO's Quality Physical Education (QPE) Guidelines, which highlight the importance of PE in promoting holistic development and lifelong engagement in physical activity (7). Educational approaches can be characterised by several fundamental principles such as accessibility, holistic understanding, integral development, individuality, experimentation and lifelong promotion (29, 30).

In recent years, theoretical advancements in PL have been notable. However, the practical application in educational contexts remains limited, and PL is still largely regarded as an emerging concept (13, 31). Consequently, empirical research remains insufficient, particularly concerning the effective mobilisation of PL principles into educational practice through intervention (13, 31, 32). Although increasingly emphasised in curriculum and policy documents, there is still limited evidence on how PL is enacted, experienced, and understood in school settings. Existing reviews underscore persistent challenges, notably the lack of a holistic approach in most interventions, which tend to prioritise the physical domain over cognitive, affective and social domains (24, 29, 32–34). This imbalance is largely associated of quantitative designs in PL research, which restricts insight into perceptions, decision-making, relationships, and emotional engagement, elements that qualitative approaches capture more effectively (24, 32, 35). Furthermore, quantitative methods have limited understanding of participants' lived experiences and the contextual factors shaping intervention effectiveness (24, 29, 33, 34, 36). While most studies and reviews follow this quantitative trend, several qualitative studies already exist but remain unsystematised. For example, Carl et al. (30), despite including mixed designs, analysed only quantitative outcomes and explicitly called for systematic qualitative syntheses to consolidate emerging evidence. A qualitative review at this stage can therefore make an essential contribution by foregrounding individual experiences and capturing complex, integrated phenomena, such as interactions with peers, teachers, and the learning environment, which are central to the phenomenological and existential foundations of PL (24).

Given this gap, the present review maps available qualitative data on school-based PL interventions to deepen understanding of how PL is developed and experienced in authentic educational contexts. Specifically, it analyses how different PL domains are fostered, the influence of pedagogical strategies, and how teachers and intervention providers shape programme quality and impact. To guide this analysis, the following research questions were formulated:

1. What qualitative evidence exist on how the different domains of PL (physical, cognitive, affective/psychological, and social) fostered in school-based interventions?
2. What pedagogical strategies are employed to promote PL, and how do they influence students' experiences and outcomes.

By synthesising this qualitative evidence, this review addresses a critical gap in the literature and offers insights to inform future pedagogical approaches and implementation strategies.

## 2 Methods

This review followed the 2020 Preferred Reporting Items for Systematic Reviews and Meta-Analyses (PRISMA) guidelines, which “can be used for original systematic reviews, updated systematic reviews or continuously updated (‘live’) systematic reviews” [(37), p.2]. However, considering that the focus of this review is qualitative data, it was decided to combine three different ways of identifying studies, as explained in the following section. This systematic review was not registered in PROSPERO or any other platform. A protocol was not prepared.

### 2.1 Procedures

The research strategy involved three stages. In the first stage, a preliminary search of previously published systematic reviews on PL was conducted, and the empirical studies included within them were analysed and extracted. In the second stage, the search protocols of the identified reviews were replicated to incorporate studies published after their respective timeframes. Finally, in the third stage, an independent search was carried out to identify additional studies not captured in the previous stages. The details of each stage are provided in the following sections:

#### 2.1.1 Stage 1—Preliminary search

This stage comprised two complementary phases. The first phase involved a preliminary search conducted to identify systematic reviews in the field of PL. This search was carried out using the EBSCOhost and Web of Science databases, using the combination of the terms “*Physical Literacy*” *AND* “*Review*” in their native search interfaces.

The search yielded four systematic reviews (24, 29–31), which were selected based on the following criteria: (i) a focus on PL; (ii) inclusion of the word “review” in the title; (iii) considered exclusively empirical studies; and (iv) did not exclusively address adult populations or those with special needs. These criteria were established to ensure the relevance of the selected studies in relation to the objectives of the present research. The empirical studies included in the selected systematic reviews were then identified and extracted for further analysis.

All four reviews adhered to PRISMA guidelines and provided transparent descriptions of their search strategies, eligibility criteria, and quality appraisal procedures. These reviews included empirical studies on PL interventions with school-aged participants, making them valuable sources for identifying potentially relevant records. Nevertheless, studies identified through previous reviews (Stage 1) and through the replication of earlier protocols (Stage 2) were not automatically included. All records retrieved across the three stages were screened using the eligibility criteria of the present review, followed by full-text evaluation and quality appraisal. Thus, earlier reviews served only as an extended search strategy to maximise sensitivity, while inclusion was determined exclusively by the criteria and appraisal methods defined in this study.

#### 2.1.2 Stage 2—Replication of the research protocols

This stage involved the replication of the research protocols from the four identified systematic reviews. The searches covered the period from the date of each review until April 2024.

For instance, the review conducted by Edwards and colleagues (24) employed a search strategy utilising Boolean logic combinations, with the following search equation: [“physical literacy” AND (measurement OR assessment OR charting OR monitoring OR evaluation OR test OR analysis OR case study OR practical OR applied OR intervention OR trial OR predictor OR correlation OR association OR relationship)]. This search was conducted in the MEDLINE (advanced search: Title/abstract) (via PubMed), Scopus (advanced search: Title/abstract and keywords) and ScienceDirect (advanced search: Title/abstract and keywords) databases. The search was replicated for the period from June 2017 (the original end date of their review's search) to April 2024. Notably, the SPORTDiscus and Education Research Complete databases were excluded from this replication due to a lack of subscription access.

In the case of the reviews by Carl and colleagues (29, 30), as they are related reviews that employ the same search protocol and databases, a single replication of the search was carried out. This replication was based on the most up-to-date version of the data (29), covering the period from November 2021 to April 2024. The authors used the following research equation: (“Physical Literacy” AND intervention OR program OR training). This research was carried out in EbscoHOST (advanced search: abstract), ProQuest (advanced search: abstract), Cochrane Central Register of Controlled Trials (CENTRAL) (advanced search: Title/abstract and keywords), PubMed (advanced search: Title/abstract), Science Direct (advanced search: Title/abstract and keywords), Scopus (advanced search: Title/abstract and keywords) and Web of Science (advanced search: Title/abstract) databases.

In the review carried out by Claudia (31), the research equation used was: (“physical education” OR “physical education pedagogy” OR “pedagogical approaches” OR “physical education strategy”) AND [“physical literacy” OR “physical literacy” AND (“active lifestyle” OR “healthy lifestyle”)] AND (children OR “school-age children” OR “primary school students” OR “secondary school students” OR “high school students” OR “teenagers”). This search was carried out in Scopus (advanced search: Title/abstract and keywords), Web of Science (advanced search: Title/abstract) and two Chinese databases (China Academic Journals Full-text Database and Taiwan Citation Index—Humanities and Social Sciences). The search carried out by the authors took place between 2011 and 2021 and the replication covered the period from January 2021 to April 2024.

Replication of the search was not performed in Chinese databases, as only English-language studies were considered in this review. The studies identified through this replication of the search protocols were subsequently integrated with the empirical studies extracted from the systematic reviews identified in stage 1.

The following databases were searched through their native search interfaces, and all retrieved records were exported directly into EndNote (version 20.2.1) using each database's automated export function, ensuring accurate reference management and facilitating the subsequent screening process.

#### 2.1.3 Stage 3—Independent search

To further address the specific objective of this review, which focused on qualitative data from PL intervention studies conducted in school settings, an independent search was conducted. The search strategy was guided by the SPIDER framework (*Sample, Phenomenon of Interest, Design, Evaluation, Research type),* which is widely recommended for qualitative and mixed-methods systematic reviews (38, 39). In this context, the *Sample* corresponded to school-aged children and adolescents; the *Phenomenon of Interest* was PL; the *Design* included structured school-based interventions or programmes; the *Evaluation* referred to participants' experiences and perceptions; and the *Research* type was restricted to qualitative approaches.

A search strategy based on Boolean operator combinations was adopted, using the following search equation: [“physical literacy” AND (School OR “Physical Education”) AND (Intervention OR Program OR Training OR “Curricular Unit”) AND (Qualitative OR Interpretative OR Interviews OR “Focus Group” OR Observation)]. The search was performed in three databases: EBSCOhost (advanced search: abstract), Scopus (advanced search: Title/abstract and keywords), and Web of Science (advanced search: Title/abstract). No date filters were applied, and the last update was in April 2024. During this stage, searches were also carried out using the databases' native search interfaces, and all records were automatically exported directly to the reference management programme.

This three-stage approach was designed to integrate existing systematisations from previous reviews, leverage relevant studies included within them that may not have been subjected to qualitative analysis, and incorporate newly published research. This specific focus on qualitative data, rather than quantitative data predominant in most existing PL reviews, was critical for exploring the nuances perspectives and lived experiences, central to the objective of this study.

### 2.2 Eligibility criteria

The eligibility criteria for the review required full-text studies on: (i) PL interventions; (ii) conducted in school settings; (iii) targeting individuals aged 5–18 years; (iv) reporting qualitative results; and (v) written in languages English, Portuguese, and Spanish. Literature syntheses, conference contributions, and theses were not considered. Naturalistic interventions without specified content or practical outcome data were also excluded.

**Figure 1:**
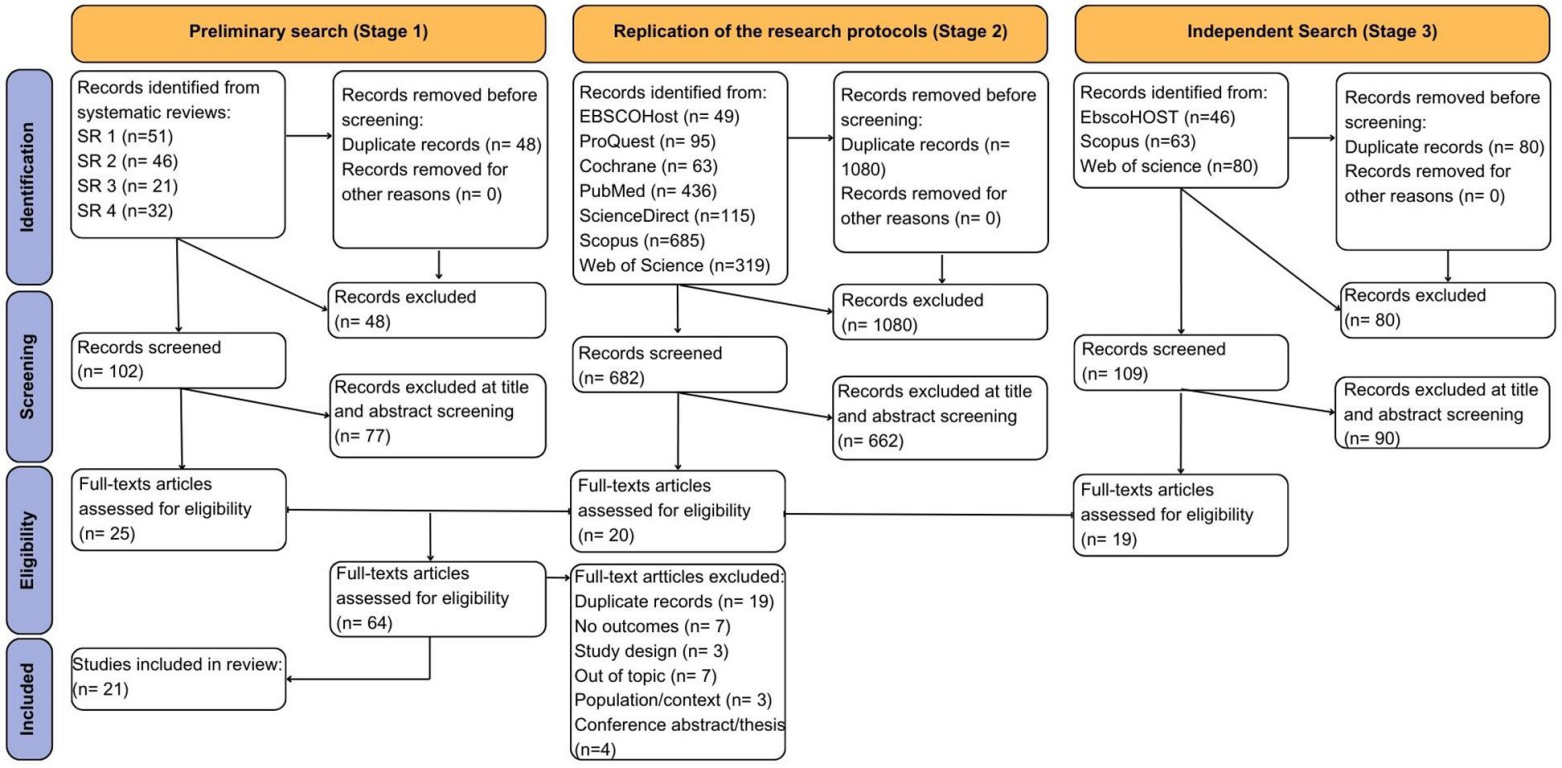
PRISMA flow diagram adapted by the authors, based on the template by Page et al. (37).

Figure 1 presents the authors' adaptation of the PRISMA flow diagram, based on the model by Page et al. (37). The search strategy was jointly developed by all four authors. The study selection process were conducted by the first author and a second reviewer (DV, TSD), who independently screened titles, abstracts, and full texts. The observed agreement rate during the selection process was 93%. Whenever uncertainties or disagreements arose regarding eligibility, a third reviewer (PB) was consulted, and final decisions were reached through discussion and consensus among the three authors.

### 2.3 Selection of studies and descriptive synthesis

The references obtained from this search were managed using EndNote software, version 20.2.1 (Clarivate Analytics). Duplicate references were first removed automatically and then manually. A screening process was carried out in three distinct phases: firstly, the studies were identified, and the duplicates of each search were managed, then the title and abstract of the studies were analysed and, finally, the full text.

#### 2.3.1 Stage 1—preliminary search

A total of 150 references resulting from studies included in the four identified systematic reviews. After removing 48 duplicates, the number of references was reduced to 102. The titles and abstracts of the 102 studies were analysed, applying the eligibility criteria. 25 studies were considered for the full text analysis.

#### 2.3.2 Stage 2—replication of the research protocol

A total of 1,762 new references resulted from the replication of the search criteria from the four identified systematic reviews in electronic databases. After removing 1,080 duplicates, the number of references was reduced to 682. The titles and abstracts of the 682 studies were analysed, applying the eligibility criteria. 20 studies were considered for full-text analysis.

#### 2.3.3 Stage 3—independent search

189 new references were considered. After removing 80 duplicates, the number of references was reduced to 109. The titles and abstracts of the 109 studies were analysed, applying the eligibility criteria. 19 studies were considered for full-text analysis.

The 64 studies considered for the full-text analysis (25 from Stage 1, 20 from Stage 2, and 19 from Stage 3) were exported to a new EndNote file, where 19 duplicate studies were removed. A full-text analysis was carried out on 45 studies.

Following this analysis, studies were excluded for the following reasons: seven studies were eliminated due to the lack of practical results (e.g., protocols not yet implemented and theoretical trials); three due to study design issue (lack of theoretical support or insufficient methodological description); seven because they were not related to the topic of PL; three due to population age or intervention context (one study did not specify participant age, another targeted children under five, and another was conducted outside a school setting, specifically in a swimming school); and four because they were conference abstracts or theses.

After the full-text analysis, 21 studies met all eligibility and inclusion criteria. The reference selection process for this review is summarized in Figure 1.

### 2.4 Data extraction and analysis

For the studies included in this review, a standardized data extraction model was used, following the approach of Carl et al. (29, 30). This model recorded information on several aspects: basic article details (authors, year of publication, country), methodology (study type, context, population, data collection instruments), intervention implementation (brief description, duration, provider), theoretical framework (definition of PL), objectives, and main qualitative findings. A comprehensive summary of this information is presented in Supplementary Table S1.

### 2.5 Quality appraisal

To ensure methodological rigour, all included studies were evaluated using the *Critical Appraisal Skills Programme* (40) Checklist for Qualitative Research. The assessment focused on the clarity of research aims, appropriateness of the methodological design, transparency in data collection and analysis, ethical considerations, and the coherence and relevance of the findings to the review objectives. A detailed CASP checklist assessment for each study is provided in the Supplementary Table S2.

All 21 included studies met quality thresholds for inclusion, although variability was noted in the depth of methodological reporting. Some studies provided limited detail regarding on analytical procedures, the role of the researcher or ethical considerations. No studies were excluded based on quality, however the appraisal informed the interpretation of findings during thematic synthesis.

Quality appraisal was conducted independently by the first author and discussed with the other three authors to enhance reliability. Discrepancies were resolved through discussion and consensus.

Based on this extraction, the 21 studies were analysed using a hybrid thematic analysis approach, combining both deductive and inductive strategies (65). The thematic analysis process involved four stages: (i) reading each article and noting key findings and conclusions related to how the intervention was conducted; (ii) compiling the findings from each article into a summary document; (iii) labelling the findings of each article with initial codes before grouping them into more generic topics; and (iv) organising these broader topics into themes.

The deductive analysis aimed to identify elements and strategies related to the four core domains of PL: physical, cognitive, affective/psychological and social. An inductive approach was them employed to identify subcategories within each domain (see Table 1), as well as recurrent themes that emerged across studies, specifically: i) challenges to program implementation; ii) and recommendations for practice. These six thematic categories provided the structure for reporting and interpreting the results of this review.

**Table 1:** Summary of Key elements and pedagogical strategies by PL domain.

Key elements and pedagogical strategies	Studies
Cognitive Domain	
Key Elements:	
Cognitive processing and knowledge construction	Alagul et al. (41); Brotoleto et al. (42); De rossi et al. (43); Farias et al. (44); Morgan et al. (45); Liu and Chen (46); Lloyd (47); Muzakki et al. (48); Ragoonaden et al. (49); Schmittwilken et al. (50); Strobl et al. (51), Woo and Lee (52)
Executive and self-regulatory skills	De Rossi el al. (43); Gavigan et al. (53); Invernizzi et al. (54); Ragoonaden et al. (49); Telford et al., (55)
Communication and expression	De Rossi el al. (43); Telford et al. (55); Invernizzi et al. (54)
Pedagogical Strategies:	
Interactive and experiential learning	De rossi et al. (43); Farias et al. (44); Gavigan et al. (53); Muzakki et al. (48); Ragoonaden et al. (49); Strobl et al. (51)
Student-Centered and autonomous learning	Farias et al. (44); Morgan et al. (45); Strobl et al. (51); Telford et al. (55); Woo and Lee (52)
Analytical and reflective thinking tasks	Alagul et al. (41); Morgan et al. (45); Woo and Lee (52); Schmittwilken et al. (50); Lloyd (47)
Physical Domain	
Key Elements:	
Overall motor skills	Anico et al. (56); Bannon (57); Bortoleto et al. (42); De Rossi et al. (43); Demetriou et al. (58); Farias et al. (44); Gavigan et al. (53); Invernizzi et al. (54); Morgan et al. (45, 48); Ragoonaden et al. (49); Telford et al. (55); Wainwright et al. (59)
Pedagogical Strategies:	
Experiential and social learning	Anico et al. (56); Bortoleto et al. (42); De Rossi et al. (43); Wainwright et al. (59); Farias et al. (44); Gavigan et al. (53); Muzakki et al. (48); Ragoonaden et al. (49)
Individualized and nurturing approaches	Bortoleto et al., (42); Bannon (57); Morgan et al. (45)
Psychological Domain	
Key Elements:	
Self-perception and agency	Anico et al. (56); Bannon (57); Bortoleto et al. (42); De Rossi et al. (43); Farias et al. (44); Gavigan et al. (53); Liu and Chen (46); Lloyd (47); Morgan et al. (45, 48); Ragoonaden et al. (49); Telford et al. (55); Woo and Lee (52); Wainwright et al. (59)
Motivation and engagement	Alagul et al. (41); Anico et al. (56); Bannon (57); Bortoleto et al. (42); De Rossi et al. (43); Demetriou et al. (58); Edwards et al. (60); Farias et al. (44); Gavigan et al. (53); Invernizzi et al. (54); Lloyd (47); Liu and Chen (46); Morgan et al. (45, 48); Ragoonaden et al. (49); Schmittwilken et al. (50); Strobl et al. (51); Telford et al. (55); Wainwright et al. (59); Woo and Lee (52)
Pedagogical Strategies:	
Structure and guidance	Anico et al. (56); Alagul et al. (41); Bannon (57); Invernizzi et al. (54); Ragoonaden et al. (49).
Learner autonomy and agency	Anico et al. (56); Bannon (57); Bortoleto et al. (42); De Rossi et al. (43); Farias et al. (44); Gavigan et al. (53); Invernizzi et al. (54); Liu and Chen (46);; Lloyd (47); Morgan et al. (45), Ragoonaden et al. (49); Schmittwilken et al. (50); Strobl et al. (51); Wainwright et al. (59); Woo and Lee (52)
Supportive learning environment	Bortoleto et al. (42); Bannon (57); Demetriou et al. (58); Invernizzi et al. (54); Liu and Chen (46); Lloyd (47); Morgan et al. (45); Telford et al., (55); Wainwright et al. (59)
Active learning and engagement	Alagul et al. (41); Anico et al. (56); Bannon (57); Bortoleto et al. (42); Gavigan et al. (53); Muzakki et al. (48); Strobl et al. (51)); Woo and Lee (52)
Relevance and Connection	Strobl et al. (51); Wainwright et al. (59)
Assessment and feedback strategies	Bannon (57); Edwards et al. (60); Farias et al. (44); Muzakki et al. (48)
Social Domain	
Key Elements:	
Interpersonal relationships and support	Anico et al. (56); Bortoleto et al. (42); De Rossi et al. (43); Gavigan et al. (53); Liu and Chen (46); Morgan et al. (45, 48); Ragoonaden et al. (49); Schmittwilken et al. (50); Telford et al., (61)
Collaboration and collective engagement	Bortoleto et al. (42); De De Rossi el al. (43); Farias et al. (44); Gavigan et al. (53); Invernizzi et al. (54); Liu and Chen (46); Morgan et al. 45, 48); Telford et al., (55)
Social and ethical awareness	Bortoleto et al. (42); Farias et al. (44); Lloyd (47); Ragoonaden et al. (49)
Pedagogical Strategies:	
Peer learning and interaction	Anico et al. (56); Bortoleto et al. (42); De Rossi el al. (43); Farias et al. (44); Morgan et al. (45, 48)
Collaborative learning and discourse	Bortoleto et al. (42); Farias et al. (44); Morgan et al. (45); Telford et al., (55)

## 3 Results

The findings are presented in two sections: Descriptive overview—outlines contextual characteristics of the included studies, such as authorship, country of implementation, target population, intervention setting, and number of participants; Thematic overview—synthesizes the results according to the six categories resulting from the thematic analysis. To ensure methodological rigour, the thematic analysis was triangulated by two independent researchers, enhancing the credibility and consistency of the identified categories.

### 3.1 Descriptive overview

Based on the design of the PRISMA flowchart, Figure 1 highlights the successive selection process from initial identification to the final 21 studies that met the eligibility criteria.

The 21 studies were authored by 81 authors. The most prolific authors in the area of research include J. Keegan, who published three studies, and K. Morgan, S. Olive, Telford, M. Telford, and S. Keegan, each of whom published two studies. There has been an increase in publications, with 11 studies published in the last four years (2024 and 2020), and 10 studies between 2019 and 2012. These 21 studies appeared in 15 different journals, with the highest number published in the Journal of *Physical Education & Sport Pedagogy* (five studies), followed by the *Journal of Teaching in Physical Education* (two studies) and *Frontiers in Sports and Active Living* (two studies).

A total of six interventions were conducted in the United Kingdom; three programs came from Canada and Germany; two from Australia, and the remaining seven interventions stemmed from Indonesia, Ireland, Italy, Korea, Portugal, Turkey and USA. The target population of the studies are primary school children in 13 interventions, elementary school (6th and 7th grade) students in five studies, secondary school students in two papers and one study considers both primary and elementary school students. With regard to the intervention context, 13 interventions reported taking place specifically in the PE context and eight took place in the school context, but without a direct link to PE classes (e.g., school sports, playground, traditional classes). Demetriou et al. (58) work was the only one that, in addition to regular schools, also considered sports-oriented schools. In the majority of the cases, the interventions were open to both genders; there was only the exception of Bannon's (57) work, which only considered female participants.

The interventions with the smallest samples (excluding potential control groups) had between seven and 10 pupils, while the largest PL interventions contained between 141 and 925 pupils. Two interventions only considered class teachers and head teachers in their population, despite the target group being young students. Telford's intervention (55, 61) led to two separate studies, both included in this review, where one reflected the students' perspectives (*n* = 318) and the other the teachers' perspectives (*n* = 14). Further details relating to the included studies can be found in the summary presented in the Supplementary Material.

In terms of qualitative data collection, the included studies employed a wide range of approaches; 12 used interviews, seven focus groups, and nine incorporated observational methods. Document analysis was also common, appearing in 14 studies through materials such as logbooks, worksheets, learning diaries, or e-portfolios. One study used photo-elicitation. This methodological diversity reflects the variety of strategies to capture participants' experiences and perceptions within school-based PL interventions.

### 3.2 Thematic overview

**Figure 2:**
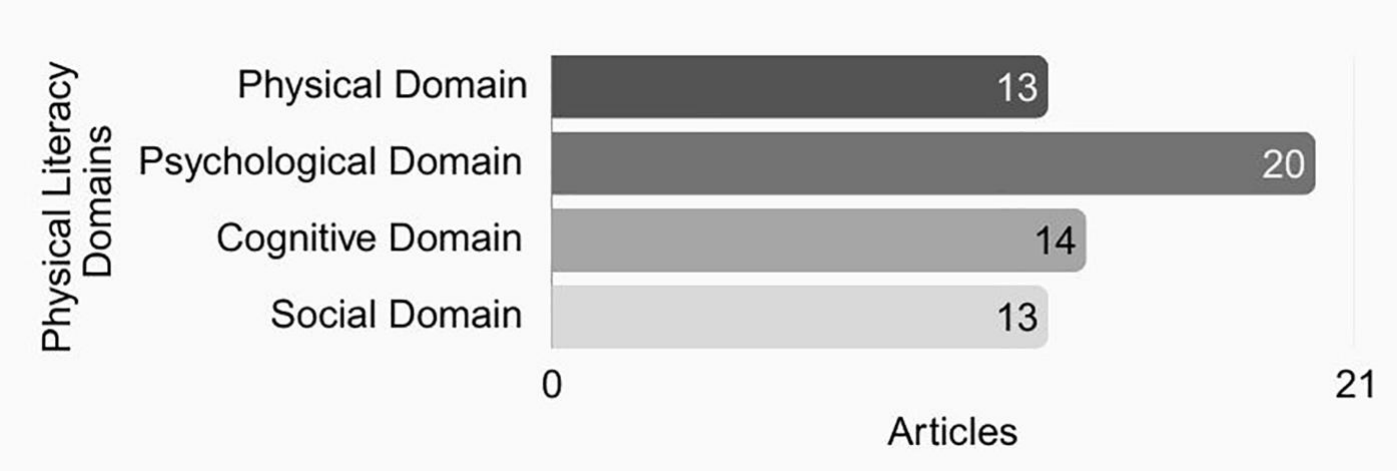
Frequency of PL domains addressed across included studies.

All interventions reported positive outcomes (see Supplementary Files) and appeared to contribute in some way to the development of students' PL. However, several studies did not explicitly address all four domains of PL in their findings, limiting the ability to assess holistic development. Figure 2 illustrates the presence of each PL domain across the 21 included studies. The psychological domain was the most frequently addressed, followed by the cognitive domain, and then the physical and social domains. Nine studies addressed all four PL domains in their findings or conclusions. Furthermore, most of the remaining studies focused on more than one domain simultaneously, pointing the interconnected and holistic character of PL in school-based interventions.

Table 1 presents a more detailed analysis of each PL domain. For each domain, the key elements highlighted in the studies are identified, along with the pedagogical strategies reported by the authors as contributing to the development of that domain. Beyond domain-specific insights, the table also reflects the interconnected nature of PL. Improvements in one domain often appear to facilitate progress in others. For example, gains in motivation and engagement (psychological domain) frequently enhanced physical competence and social interaction (observed in 11 studies). Motivated students tended to participate more actively, which not only improved their motor skills but also encouraged peer collaboration. Moreover, several pedagogical strategies were found to support the development of multiple domains simultaneously, further reinforcing the holistic character of PL.

Although pedagogical strategies may vary across age groups and educational contexts, the available qualitative evidence did not support a meaningful stratified analysis. Over half of the included studies (13 out of 21) were conducted in primary school, with comparatively fewer studies in older age groups and only two focused on secondary education. Moreover, most studies did not report domain-specific findings by age or context.

Despite this heterogeneity, several pedagogical strategies appeared to exert a similar effects across educational levels (Table 1). For instance, interactive and experiential learning was consistently linked to the development of cognitive PL domain in primary, elementary, and secondary samples (43, 44, 51, respectively). A similar pattern emerged for student-centred and autonomous learning, reported as beneficial across different age groups (44, 45, 52). Additional examples are provided in Table 1.

Finally, the following table (Table 2) summarises the themes that emerged across the studies. The main challenges identified include inconsistencies in the implementation of the programs, which can arise from factors such as time limitations, spatial constraints, curriculum issues, or teacher resistance and difficulties. Additionally, recommendations for practice primarily focus on the importance of continuing professional development and providing opportunities for collaboration.

**Table 2:** Emerged themes across the studies.

Emerged themes	
Challenges to the implementation of programs	Recommendations for practice
Operational constraints (Curriculum, space, weather, time…)	Ongoing teacher training, support, and peer collaboration
Challenges in applying theoretical concepts	Foster mastery climates with personal goals, flexible pacing, and peer-based collaboration
Resistance to changing traditional practices	Encourage shared decision-making between students and adults

## 4 Discussion

### 4.1 The interconnected nature of PL domains

This systematic review aimed to map the qualitative data available on school-based interventions related to PL to understand how PL is developed and experienced within authentic educational contexts. In line with the review's aims, the discussion is organised to address the two research questions: how the four domains of PL are supported through intervention practices, and which pedagogical strategies and learning environments facilitate this development. Given the holistic nature of PL, this review adopts a structured yet interconnected approach to data analysis. This interconnectedness reflects the foundational assumptions of PL, which define it not merely as a set of skills but as a unified construct that fosters lifelong engagement in physical activity (26, 28).

The lived experiences reported in the studies reviewed highlight the interplay of PL and the strong interconnections between its domains. These findings align with existing literature, as several authors have emphasised the potential for these domains to interact dynamically and mutually develop [e.g., (13, 20, 62)]. However, this review also acknowledges the value of identifying patterns across individual domains, as this can provide concrete insights to inform pedagogical design and development of interventions. This perspective is consistent with a more integrative approach, such as that proposed by Young et al. (21), which aims to connect philosophical coherence with practical guidance in educational settings.

Extending this integrative perspective into practice, the findings reveal that various domains often develop simultaneously, supported by pedagogical strategies that go beyond singular categories. This interdependence is also evident in the qualitative findings across studies. For instance, Alagul and colleagues (41) suggest that teaching psychomotor skills alongside cognitive activities enriched overall learning outcomes. Anico et al. (56) argue that participating with friends, in addition to fostering the social domain, positively influenced the participants' willingness and motivation to participate, showing a direct relationship with the psychological domain. Furthermore, the same authors point out that allowing students to set their own pace not only boosted their confidence (psychological domain) but also enhanced their physical abilities (physical domain).

Telford et al. (55) explored activities that fostered both autonomy and teamwork, encouraging children to develop new skills and collaborate effectively. While these two strategies might initially appear to be contradictory, Bortoleto et al. (42) noted that promoting autonomy enables students to learn at their own pace while receiving support from their peers. This approach enhances opportunities for meaningful collaboration. Furthermore, these strategies not only improve social interaction but also encourage students to explore new skills, often leading to increased creativity, engagement, and motor involvement. These elements go beyond social domain, also intersecting with cognitive, psychological, and physical domains.

In Muzakki and colleagues' (48) study, the use of peer-teaching supported not only resulted in better relationships among students (social domain) but also their engagement in the subject matter (psychological domain), which in turn influenced the cognitive domain. The authors highlight that the act of teaching peers required students to engage more deeply with the subject, leading to better knowledge retention and conceptual understanding. Similarly, in Lloyd's (47) study, students described increased awareness of their bodily sensations during climbing activities, revealing a strong connection between physical engagement and psychological reflection. These interrelationships were also visible in domain-specific analysis. For instance, pedagogical strategies such as peer teaching or personalised feedback emerged as contributors across all domains of PL. A single strategy, while perhaps intended to develop one specific aspect of PL, often had broader benefits. For example, peer teaching supported not only social learning and collaboration but also boosted confidence, motor skills, and knowledge construction. Likewise, personalised feedback was linked to improvements in the physical, cognitive, and psychological dimensions (46, 52, 57).

Still within this holistic perspective, several pedagogical features consistently appeared across interventions and seem central to promoting integrated PL development. These included: a self-referenced approach to learning (45, 50), non-judgmental learning environments focused on effort and progress (45, 48, 50), and student-centered methodologies (44, 48, 55). In all these aspects, the prominent role of the student and the respect for their individuality are common. These findings align with the results of Claudia's (31) systematic review, which had already emphasised the importance of adopting student-centered pedagogical approaches. Participants value having time and space to learn at their own pace, being able to set goals that are relevant to them and prefer to be compared with themselves (their progress), rather than being compared with others (peers or national averages) (42, 57, 60). These aspects seem to be critical for fostering youth engagement in interventions designed to develop PL. A particular noteworthy feature is the use of ipsative assessment, as reported in studies by Bannon (57) and Edwards et al. (60). This approach evaluates a student's current performance against their previous achievements, emphasising individual progress rather than comparisons with peers or external standards.

This type of formative, student-focused assessment is particularly relevant in PE, where tracking personal growth can support motivation, self-esteem, and accountability (63, 64). Although traditionally discussed in terms of its effects on the psychological domain, this strategy seems to extend more broadly, promoting cognitive reflection, motor competence, and even social dynamics such as collaboration and inclusion.

Although all reported positive outcomes, a trend consistent with previous systematic reviews of PL interventions (24, 30–32), only nine of the twenty-one studies explicitly addressed all four PL domains. These findings underscore a persistent concern: empirical research remains limited and insufficient in demonstrating a coherent and holistic mobilisation of PL principles in practice. This is particularly noteworthy given the assumption that qualitative methodologies are well suited to capturing complex, integrated, and relational phenomena central to PL's phenomenological and existential foundations (24). In theory, such approaches should facilitate a more holistic representation of PL development. Yet, our results suggest otherwise: similar to quantitatively oriented reviews, most interventions did not present explicit evidence across all four domains. One plausible explanation lies in the conceptual ambiguity that surrounds PL (16). Interventions often reflect authors' philosophical stance—some adopting idealist perspectives and others more pragmatic interpretations (21). Researchers aligned with idealist views frequently favour qualitative designs (24), but because these approaches are rooted in phenomenological and existential foundations, they may avoid systematically operationalising each domain. Consequently, domains may indeed be present in the data but only implicitly, limiting interpretability.

The findings of Young et al. (21) and Lower-Hoppe et al. (23) offer a valuable theoretical lens for interpreting the differing perspectives on PL development. On one hand, the evidence supports the holistic and relational view of PL, consistent with the idealist perspectives. On the other hand, a pragmatic, domain-specific approach emphasises the importance of practical applicability. That said, it is evident that a pragmatic domain-specific approach is necessary for designing pedagogical interventions that effectively address all domains. Therefore, this review endorses an integrative approach: the developing pedagogical models that are philosophically aligned with PL holistic nature while remaining practical and actionable for teachers.

The literature has long encouraged creative and non-traditional approaches to PL interventions (24, 36), and this review indicated that several studies are moving in this direction. However, it remains essential that, researchers, regardless of philosophical orientation, explicitly address and report all learning domains. Adopting structured reporting templates, such as the PLIRT protocol (65), could substantially enhance consistency and transparency in future PL intervention research.

### 4.2 Navigating each domain: A deep dive into PL

The qualitative research shows a greater emphasis on results in the psychological domain than previous quantitative reviews (30, 32). This domain was represented in 20 out of 21 analysed articles. Improvements in psychological domain elements such as motivation, engagement, and confidence are strongly represented in observations and participants' narratives. These results suggest that qualitative data help us to understand how students and teachers get involved in interventions and how specific strategies (such as personalised feedback, autonomy-supportive teaching, and non-judgmental environments) impact their satisfaction, confidence, self-esteem, and motivation to get involved in physical activities. Data revealed that goal-setting and self-paced challenges were effective in boosting students' self-confidence and intrinsic motivation. For instance, students who engaged in ipsative assessment and activities that promoted autonomy and choice in tasks exhibited greater engagement, satisfaction, and commitment to improvement (56, 57, 59).

The physical domain was not as prominent as it typically is in quantitative interventions (30, 32). 13 out of 21 interventions reported improvements. This may be because in participants' narratives and observations, we can only gauge students perceived motor competence rather than measuring it objectively. As a result, this domain tends to be less present than in other studies. Despite this, key findings indicated improvements in fundamental motor skills, including object control, locomotion, and balance (42, 43, 53, 55, 58). Daily PE sessions enhanced coordination and basic movement functions (42, 58). Individualised and nurturing approaches, such as ipsative assessment—where students track their personal progress rather than comparing themselves to others—further promote skill acquisition (42, 57). Additionally, experiential and social learning in open environments appears to favour the development of young people's physical skills by exposing them to various movement situations (43, 44, 48, 59).

In the cognitive domain, 14 interventions reported positive outcomes. Like the physical domain, the cognitive domain is also more easily “measured” through tests and questionnaires. In any case, it was expected that the students would report more frequently on their learning, both in relation to the intervention content itself and the knowledge that could later support their participation in physical activities throughout life. We highlight six interventions that reported progress in knowledge and understanding (41, 44–46, 48, 51). These results can be interpreted as stemming from interventions that incorporated strategies to develop students' cognitive skills, including both task-integrated approaches—where cognitive abilities were engaged during physical performance, and complementary reflective activities such as writing, analysis, and synthesis tasks designed to encourage autonomy and deeper understanding.

As for the social domain, 13 interventions reported improvements. Key findings indicated improvements in social domain elements, such as inclusion, empathy, respect, social justice, and collaboration. Some strategies like peer support and cooperative tasks, were implemented to foster teamwork, empathy, and interpersonal skills. However, the strategy that ultimately stood out was peer teaching, it seems to be a very effective approach in fostering the social domain. This strategy was observed in the studies by Muzakki et al. (48), De Rossi et al. (43) and Farias et al. (44), as well as in the study by Bortoleto et al. (42), where, after an initial phase more centred on the teacher/instructor, naturally students with a strong mastery in some circus disciplines (e.g., unicycle, ball-roller, etc.) acted as mentors in the class, helping the teacher and motivating their classmates with new challenges. In addition, in this case, they played an important role in the inclusion of classmates with different physical or cognitive abilities. In this domain, what was frequently present in the participants' speeches was “Enjoying playing/participating with friends”, rather than deeper connections related to inclusion, empathy, respect, equity and social justice.

Something that may have influenced this is the way the social domain was studied and how data were collected in the various interventions. For example, the construction of interview scripts or the observations conducted, could have been more focused on other domains rather than on the social domain. Also, the age of the participants (14 of the 21 studies were carried out in primary school) may have contributed to more superficial and less in-depth connections, as in the example given above. This consideration could also apply to other domains.

When examined domains individually, the findings reinforce patterns documented in the broader literature. Persistent challenges remain, as most interventions fall short of achieving a holistic approach to PL, with marked imbalances across domains (24 29, 32–34). A distinctive contribution of this review is the prominence of the psychological domain in studies using qualitative methods, contrasting with quantitatively oriented reviews, where the physical domain dominates.

Our findings align with previous research: despite qualitative also struggles to capture PL's holistic nature, it tends to foreground domains often underrepresented in quantitative assessments (24, 32, 35). In particular, psychological, social, and cognitive dimensions emerge more clearly through qualitative inquiry.

### 4.3 Beyond domains: the pivotal role of teachers in PL development

One of the most significant findings from the analysis is the pivotal role that teachers and intervention providers play in shaping both the quality of implementation and the overall student experience. This influence is so impactful that the majority of the interventions emphasise the importance of ongoing professional development and teacher support [e.g., (42, 53, 60)] and peer collaboration opportunities (42, 60, 61)—community of practice through “WhatsApp” (42).

The impact of teachers can be seen in several areas: in how well they understand the concept of PL (56, 60); in their ability to create inclusive environments that foster motivation and autonomy (43, 45, 54, 56); and in the extent to which they integrate opportunities for PL across the broader school curriculum (58).

In addition to grasping the overall concept of PL, it is essential for teachers to develop a thorough understanding of its domains and to be well-versed in pedagogical strategies that enhance their development. This domain-specific understanding enables teachers to harness the interdependence among the domains and choose strategies that promote multiple aspects of PL simultaneously. Notably, the most effective interventions are those that recognise the individuality of students and prioritise their voices and agency, placing the learner at the centre of the learning process.

Implementing a student-centered approach in education requires a significant shift away from traditional, teacher-centered practices, and it is grounded in constructivist principles (66, 67). However, many teachers find this transition challenging. Several studies included in this review highlighted that some teachers resist changing long-established teaching methods, resulting in inconsistencies in the implementation of PL programmes (51, 54, 61). As the literature highlights, adopting a student-centred pedagogy is not a straightforward process (68, 69); it requires the ability to share power between teachers and students, as well as mastery of a range of pedagogical approaches (70, 71). Additionally, including student teachers and pre-service teachers in PL interventions present both opportunities and challenges. Invernizzi et al. (54) noted issues related to the lack of experience and difficulties like conflict management. However, these students often come without preconceived notions, which positions them as the future of physical education. This context allows PL to play an important role in their professional training and development. Therefore, teacher education programs should emphasise the development of the concept of PL and incorporate it into the training of their students.

## 5 Conclusions and limitations

The findings of this review challenge the common assumption that PL interventions predominantly enhance the physical domain, as suggested in prior reviews (29, 32, 34). The psychological domain was clearly the most prominent in the interventions analysed. This study offers a novel contribution by focusing specifically on qualitative data, thereby complementing previous reviews that have relied mainly on quantitative approaches. This allows for a deeper pedagogical interpretation of how PL is fostered in school contexts and complements existing literature with rich, practice-oriented insights.

Findings suggest that flexible, inclusive approach, attentive to individual learning goals and supported student-centred methodologies and non-judgemental environments, foster greater engagement in PL interventions. Teachers also reported positive benefits for their own pedagogical practices, while emphasising the need for ongoing professional development and collaborative exchange. However, the limited number of interventions explicitly addressed all four PL domains constraints conclusions about holistic development. Future research should employ qualitative designs that explore how students and teachers experience and interpret PL interventions, providing deeper insight into processes that support the integrated development of PL (47, 52).

Despite the findings presented, we acknowledge several important limitations in this study. First, only studies published in English, Portuguese, and Spanish were included, whilst no previous review has covered as many different language publications, we may have missed potential interventions conducted and written in other languages. Second, we focused solely on published articles, overlooking potentially valuable contributions from theses, conference proceedings, and other sources. Third, the studies included in the review varied in the type of intervention: some were conducted directly with students, while others targeted teachers who then implemented the interventions with their students. This distinction was not discussed in the analysis, which may introduce some bias. Fourth, this review did not analyse the effectiveness of pedagogical strategies by participant age or specific implementation context, primarily due to substantial heterogeneity across studies. Variations in school levels, cultural settings, and instructional environments made stratified analysis unfeasible and limited direct comparisons. Consequently, the findings should be interpreted as guiding principles for future interventions, rather than definitive evaluations.

Moving forward, research would benefit from clearer reporting of PL domains, greater methodological consistency, and qualitative designs that more explicitly capture integrated development across diverse educational contexts.

## Data Availability

The original contributions presented in the study are included in the article/Supplementary Material, further inquiries can be directed to the corresponding author.
