# Supplementary material for: Exploring physical literacy in school contexts: a systematic review of qualitative evidence
Source: Front Sports Act Living. 2025 Dec 17;7:1713780. doi: 10.3389/fspor.2025.1713780 (PMC12753885; doi:10.3389/fspor.2025.1713780)
Supplement: Supplementary file 1 [file Table1.docx]

Supplementary Material

# Supplementary Table 1: Summary of studies

| Study Authors (year) / Countries | School context / Population (Age) | Objectives | Intervention (Short description) | Length / Frequency / Duration | Definition(s) of physical literacy | Type of study / Assessment | Main findings |
| --- | --- | --- | --- | --- | --- | --- | --- |
| Alagül et al. (2012) / Turkey | 7th-grade students | Evaluate cognitive learning in dance using Bloom’s taxonomy, which was taught with a PL perspective. | Implementation of a dance unit in PE classes with a focus on PL, integrating cognitive and physical domains. | 4 weeks, 80-minute sessions | No holistic definition - Physical and Cognitive domains only | Action research - case study / Student reflections, participant observation and questionnaire (Bloom’s Taxonomy) | Students were initially sceptical but gradually became more engaged. By the end of the unit, enthusiasm for both movement and learning had increased, with some students independently seeking dance-related content outside school. |
| Anico et al. (2023) / UK | Primary school students (3rd – 6th class) | Explore teachers’ perspectives on pupils’ experiences of participating in Barnet's Golden kilometre and its potential contribution to PL development. | Structured run/walk program integrated into the school curriculum to promote PL. | 1 km per day every school day, 5x a week | IPLA (2017), holistic concept | Qualitative study / semi-structured interviews | The intervention was beneficial for students who could self-regulate their pace, highlighting the role of autonomy in PL development. |
| Bannon (2013) / UK | 11-12 years old female students | Change the students' attitudes towards physical education and, more specifically, the “Bleep” test. | Application of diverse teaching strategies aimed at enhancing students' motivation, competence, and appreciation of movement within PE classes. | Not specified | Whitehead (2010), holistic concept | Case study/questionnaires, interviews, fitness testing and reflective journals | Initially, students showed low engagement, but implementing alternative instructional strategies improved motivation and participation in PE, and their results in the Bleep test. |
| Bortoleto et al. (2022) / Canada | Primary school students | Explore the implementation and impact of circus arts instruction in PE classes. | Use of circus arts activities as a pedagogical approach to foster engagement, inclusivity, and motor skill development in PE settings. | 16 hours over 2 weeks | Canadian consensus statement (ParticipACTION et al. 2015), holistic definition | Multiple case study / Participant observation, informal conversations, field notes | Circus-based PE activities created an inclusive environment, fostering engagement and improving students' motor competence and confidence. |
| De Rossi et al. (2015) / UK | Primary school students (aged 10-11) | Investigate the role of active play in promoting PL. | Investigation of unstructured and semi-structured active play opportunities in promoting PL from a child’s perspective. | Two hours of PE classes per week, and plays outside school | Whitehead (2010), holistic definition | Case study, photo elicitation and focus groups | Free play, both structured and unstructured, can improve PL by providing children with opportunities to explore their environments, develop their motor skills and engage in problem-solving. Children’s improve their self-esteem, self-confidence and motivation. |
| Demetriou et al. (2018) / Germany | Primary school students | Assess the impact of sports-oriented primary school on students’ PL and cognitive performance. | Integration of sports-focused curriculum within primary schools to develop PL and cognitive performance. | 90-min physical education lessons, 5x a week, active recess, active breaks in the classroom | Whitehead (2010), holistic definition | pilot study - non-randomised controlled trial/ interviews, curriculum analysis, physical fitness tests and questionnaires | Students exhibited modest gains in motor skills and attitudes toward physical activity, but the intervention did not lead to significant cognitive improvements. Small positive effects in PL can be attributed to the sports-oriented school. |
| Edwards et al. (2019) / South Wales, UK | Primary school students (aged 10-11) | Explore how the aforementioned professional programme modified teachers’ knowledge and operationalisation of PL. | Training program designed to improve teachers' knowledge and application of PL (PDPL programme) in their teaching practice. | 6 months, 2x week, 60 min sessions | IPLA (2016), holistic definition | Qualitative study / structured observations, reflections, and semi-structured interviews | The PD programme had an impact on teachers‘ knowledge and operationalisation of PL. The incorporation of PL principles into PE resulted in sustainable changes in teachers’ practice. |
| Farias et al. (2020) / Portugal | Grade seven school children (aged 12-14) | Examination of students’ curricular experience of Sport Education and how it transformed their subsequent PL in PE and the wider context of their lives. | Implementation of a year-long Sport Education model emphasising student autonomy, social engagement, and skill development in PE. | 1 school year, 2x week, 45-90 min sessions | IPLA (2017), holistic definition | Participatory case study with retrospective evaluation / semi-structured individual interviews, focus group, retrospective survey | A year-long Sport Education curriculum inspired a transformation in the students’ PL, which built a positive attitude toward PE practice. Three years later, the PL attributes of movement competence and confidence, and disposition to engage in empathetic social interactions developed in the program sustained the active commitment of the participants to pursue equitable and meaningful physical activity experiences to enrich their lives. |
| Gavigan et al. (2023) / Ireland | Primary school students (aged 6-10) | Assess the implementation, feasibility and effectiveness of the MWBW intervention in primary schools. | School-based intervention (MWBW Intervention) targeting the development of PL through fundamental movement skills and engagement in physical activities. | Eight-week period of three main components: (i) Two 30-min FMS-based PE classes per week. (ii) 5–10-min Active Classroom activities performed at least once daily. (iii) Home Activity Sheets completed once per week | Whitehead (2013), holistic definition | Case study / Focus groups, questionnaires and reflections | The intervention resulted in improvements in students' fundamental movement skills, confidence, behaviour and parental involvement. Increased teacher engagement in promoting PL. |
| Invernizzi et al. (2019) / Italy | Primary school students (10 yo avrg) | Compare the effects of an integration of MTA based on active reflection to standard PE practice (S-PE) on some essential features of PL. | Application of various teaching styles and active reflection in the development of PL in primary school children. | 12 weeks, 2h week | Whitehead (2010), holistic concept | Experimental study - Cluster randomised controlled trial / semi-structured interviews and video analysis; Fitness Tests and questionnaires | MTA approach, with its emphasis on variety and learning new skills, was able to capture students' interest and motivation more effectively. The MTA approach, by providing a more dynamic, interactive and reflective learning environment, promotes the development of motor, social and cognitive skills, crucial elements of PL, more effectively than the traditional approach. |
| Liu & Chen (2022) / USA | Middle school students (6th and 7th grade) | Characterise PL development and capture PL journeys in the context of receiving an SDT-guided pedagogical workshop. | PE program based on self-determination theory, aiming to characterise and capture students' PL developing trajectories. | 8 weeks, 4 workshop sessions, 20-30 min session | Whitehead (2013), holistic definition | Pilot intervention study - focus group interviews, observation notes, worksheets, CAPL-2 | Intervention was particularly effective for students with initially low PL, enhancing their frequency of participation in physical activities, motivation, confidence, and movement competence. Positive change in awareness of physical, cognitive, affective and behavioural barriers. |
| Lloyd (2016) / Canada | One, five, seven, eight, nine-grade students | Explore a conceptual shift from mechanism, the dominant ‘body-as-machine’ paradigm, to vitalism, the philosophical phenomenological tenets of PL upon which the PE curriculum is based. | Implementation of the “JungleSport” program in a school context. The program is described as an adventure-based form of learning. Students interact with obstacle courses, vertical challenges such as fixed lines, climbing walls, bouldering walls, and cargo nets. | Not specified | Whitehead (2010), holistic concept | Case study / phenomenological observations, group interviews, journal entries | Students engaged in alternative PE programs developed a broader appreciation for movement experiences, emphasising emotional and cognitive aspects. The study concludes that to truly cultivate PL, educators need to adopt a perspective that goes beyond function and form to encompass feelings, imagination, and the holistic nature of the movement experience. |
| Morgan et al. (2013) / Walles | Primary school students (aged 10-11) | Investigate the effects of a mastery motivational climate on the development of PL in Primary PE. | Intervention emphasising mastery motivational climate, on the development of PL in Primary PE. Climate was fostered through the TARGET model in PE classes. | 3 PE classes | Whitehead (2012), holistic concept | Case study / Questionnaires, semi-structured interviews, compartmental observation, diary recordings | Mastery-oriented teaching approaches can improve the PL journey of primary school students. Resulted in increased student motivation, enjoyment and perceived competence in physical education settings. |
| Muzakki et al. (2023) / Indonesia | Pencak silat athletes (elementary school, aged 11-12) | Investigate the effect of the peer teaching model on improving the PL of pencak silat athletes. | Use of the peer teaching model in martial arts training to support PL development among elementary school athletes. | 2 months, 3 times a week, 1h sessions. | Liu & Chen (2021), holistic concept | Experimental study / Interviews, fitness tests and questionnaires | Most of the athletes expressed positive perceptions of the peer teaching method, highlighting its benefits in improving their PL, particularly in motor skills, knowledge and understanding, and motivation and self-efficacy. |
| Ragoonaden et al. (2012) / Canada | Primary school students (aged 6-7) | Determine if Yoga, as a daily physical activity (DPA), promotes elements of PL in primary school children. | Incorporation of yoga practices, as a daily physical activity, to develop PL through mind-body awareness and movement exploration. | 1 week, 5 sessions, 30 min | Whitehead (2007), holistic concept - but the intervention only addressed characteristics 5 and 6 of Whitehead's (2007) PL. | Pilot study/journal responses, a semi-formal interview, group discussion, and observation | The results suggest that the regular practice of Yoga, in an educational environment and framed by Integral analysis, can promote the holistic development of students, including physical, emotional, social and academic aspects. The yoga sessions had an observable impact on Whitehead’s (2007) fifth and sixth PL characteristics. |
| Schmittwilken et al. (2024) / Germany | Primary school students (aged 8-9) | Describe the process of a female pedagogue initially familiarising herself with the PL concept and its implementation opportunities for the school setting, and retrace the process of developing and refining a PL-driven intervention for extracurricular physical education. | Pilot evaluation of extracurricular school activities designed to enhance PL through participatory engagement and autonomy-supportive teaching. | 14 sessions, 60-90 min | Sport Australia (2019), holistic concept | Self-study design/session protocols, discussions, observations, group interviews | Findings suggest that teachers' pedagogical stance and classroom organisation significantly influence the effectiveness of PL-focused extracurricular activities. A safe and trusting environment is fundamental to the success of the intervention. Teachers must have access to adequate training and support to implement the LF concept effectively. |
| Strobl et al. (2020) / Germany | Secondary school students (7th-10th grade) | Develop, implement and evaluate effective and feasible measures addressing students’ health-related knowledge and understanding in PE via a participatory approach. | One-year participatory intervention involving students and teachers in co-designing PE lessons to foster students' understanding of health and movement. | 1-year program | Whitehead (2013), holistic concept | Experimental study - Participatory research / written documentary technique, questionnaire | Participatory planning process involving teachers, principals, students and scientists is an adequate approach to conceptualise and implement evidence-based teaching methods within the school setting, which helps to increase students’ HKU. |
| Telford et al. (2021a) / Australia | Primary school students (Grade 5) | Evaluate the impact of the PEPL approach on the development of PL in primary school students. | School development approach with peer-coaching and mentoring for PE teachers (PEPL) The PEPL intervention consisted of a qualified PE teacher working with generalist class teachers to improve their skills and confidence in teaching PE. | 33 weeks, one additional PE class per week and four activity sessions (15–40 min) in the school yard | Whitehead (2013), holistic concept | Action research with a randomised cluster, accelerometry, motor test, questionnaires, focus groups, and interviews | Students who participated in the PEPL intervention exhibited improved object control skills, a trend towards more MVPA during school hours and increased confidence and motivation for physical activity. Decrease in perceived sporting competence. |
| Telford et al. (2021b) / Australia | Primary school students (Grade 5) | Evaluate the implementation, acceptability and impact of teacher delivery of PE as part of a multicomponent PEPL approach. | School development approach with peer-coaching and mentoring for PE teachers (PEPL) The PEPL intervention consisted of a qualified PE teacher working with generalist class teachers to improve their skills and confidence in teaching PE. | 33 weeks, one additional PE class per week and four activity sessions (15–40 min) in the school yard | Whitehead (2013), holistic concept | Action research with a randomised cluster / PEPL coach logbook, interviews, observation tool (SOFIT) | The PEPL approach can be an effective intervention to improve the delivery of PE, increase opportunities for physical activity and promote a positive school culture towards physical activity in primary schools. Teachers involved in the PEPL professional development program showed greater confidence in delivering PE lessons and promoted more physical activity opportunities. |
| Wainwright et al. (2018) / Wales | Primary school students (aged 5-6) | Explore the Foundation Phase as a naturalistic intervention and examine its contribution to the development of PL. | Implementation of a play-based curriculum for young children to support the development of students' PL. | Not specified | Whitehead (2016), holistic concept | Non-randomised controlled trial, motor test, video and participant observation, field notes, questionnaires | Children in the play-based curriculum showed improvements in motor skills and high levels of engagement in physical activities. The movement-rich nature of the Foundation Phase, together with its child-centred approach, may have contributed positively to pupils' beliefs about their physical abilities and their willingness to engage in physical activity. Domains such as social and cognitive are not addressed. |
| Woo & Lee (2022) / South Korea | Secondary school students | Explore features of student participation in an e-portfolio for charting PL and its impacts on student learning. | Implementation of an e-portfolio system to enable students to document, reflect on, and monitor their PL progression over time. | 10 week, 2 PE classes | Whitehead (2019), holistic concept | Case study / Interviews, e-portfolio artefacts | The use of e-portfolios in PE helped students develop a reflective approach to physical literacy, improving motivation and enabling targeted teacher support. The effectiveness of using e-portfolios to record  journeys depends largely on the students' level of involvement and self-reflection. |
