# Supplementary material for: Exploring physical literacy in school contexts: a systematic review of qualitative evidence
Source: Front Sports Act Living. 2025 Dec 17;7:1713780. doi: 10.3389/fspor.2025.1713780 (PMC12753885; doi:10.3389/fspor.2025.1713780)
Supplement: Supplementary file 2 [file Table2.docx]

Supplementary Material

# Supplementary Table 2: Critical Appraisal – CASP Checklist for Qualitative Research

| Authors | Study Design (Qualitative data) | CASP - Critical Appraisal Skills Programme Checklist For Qualitative Research | | | | | | | | | |
| --- | --- | --- | --- | --- | --- | --- | --- | --- | --- | --- | --- |
|  |  | A1 | A2 | A3 | A4 | A5 | A6 | B7 | B8 | B9 | C10 |
| Alagül et al. (2012) | Mixed (Student reflections, participant observation) | Y | Y | Y | Y | Y | Y | CT | CT | Y | Y |
| Anico et al. (2023) | Qualitative | Y | Y | Y | Y | Y | Y | Y | Y | Y | Y |
| Bannon (2013) / UK | Mixed (Interviews, reflective journals) | Y | Y | Y | Y | Y | Y | CT | CT | Y | Y |
| Bortoleto et al. (2022) | Qualitative | Y | Y | Y | Y | Y | Y | Y | Y | Y | Y |
| De Rossi et al. (2015) | Qualitative | Y | Y | Y | Y | Y | Y | Y | CT | Y | Y |
| Demetriou et al. (2018) | Mixed (Interviews, document analysis) | Y | Y | Y | Y | Y | Y | Y | N | Y | Y |
| Edwards et al. (2019) | Qualitative | Y | Y | Y | Y | Y | Y | Y | Y | Y | Y |
| Farias et al. (2020) | Qualitative | Y | Y | Y | Y | Y | Y | Y | Y | Y | Y |
| Gavigan et al. (2023) | Qualitative | Y | Y | Y | Y | Y | Y | Y | Y | Y | Y |
| Invernizzi et al. (2019) | Mixed (Interviews) | Y | Y | Y | Y | Y | Y | Y | Y | Y | Y |
| Liu & Chen (2022) | Mixed (Focus groups) | Y | Y | Y | Y | Y | Y | Y | Y | Y | Y |
| Lloyd (2016) | Qualitative | Y | Y | Y | Y | Y | Y | Y | Y | Y | Y |
| Morgan et al. (2013) | Mixed (Interviews) | Y | Y | Y | Y | Y | Y | Y | Y | Y | Y |
| Muzakki et al. (2023) | Mixed (Interviews) | Y | Y | Y | Y | Y | CT | Y | Y | Y | Y |
| Ragoonaden et al. (2012) | Qualitative | Y | Y | Y | Y | Y | CT | Y | Y | Y | Y |
| Schmittwilken et al. (2024) | Qualitative | Y | Y | Y | Y | Y | Y | Y | Y | Y | Y |
| Strobl et al. (2020) | Mixed (Written documentary technique) | Y | Y | Y | Y | Y | Y | Y | Y | Y | Y |
| Telford et al. (2021a) | Mixed (Focus groups) | Y | Y | Y | Y | Y | Y | Y | Y | Y | Y |
| Telford et al. (2021b) | Mixed (Interviews, coach logbook) | Y | Y | Y | Y | Y | Y | Y | Y | Y | Y |
| Wainwright et al. (2018) | Mixed (Participant and video observation, field notes) | Y | Y | Y | Y | Y | Y | Y | Y | Y | Y |
| Woo & Lee (2022) | Qualitative | Y | Y | Y | Y | Y | CT | CT | Y | Y | Y |

Note. Y = Yes; N = No; CT= Can’t Tell.
